# Are resident handlings in eldercare wards associated with musculoskeletal pain and sickness absence among the workers? A prospective study based on onsite observations

**DOI:** 10.5271/sjweh.3979

**Published:** 2021-10-31

**Authors:** Leticia Bergamin Januario, Svend Erik Mathiassen, Matthew L Stevens, Andreas Holtermann, Gunnar Bergström, Reiner Rugulies, Kristina Karstad, David M Hallman

**Affiliations:** 1 Department of Occupational Health Sciences and Psychology, Centre for Musculoskeletal Research, University of Gävle, Gävle, Sweden; 2 National Research Centre for the Working Environment, Copenhagen, Denmark; 3 Unit of Intervention and Implementation Research for Worker Health, Institute of Environmental Medicine, Karolinska Institutet, Stockholm, Sweden; 4 Department of Public Health, University of Copenhagen, Copenhagen, Denmark; 5 Department of Psychology, University of Copenhagen, Copenhagen, Denmark

**Keywords:** healthcare, latent profile analysis, longitudinal study, patient handling

## Abstract

**Objectives:**

We aimed to identify eldercare wards with different types of resident handling characteristics (‘phenotypes’) and determine the prospective association between these characteristics and musculoskeletal pain and sickness absence among workers during a one-year follow-up.

**Methods:**

Our study was based on the DOSES cohort, including 467 workers at 103 eldercare wards. At baseline, resident handlings were assessed using onsite observations. Workers’ self-reported musculoskeletal pain and sickness absence were assessed during the following year using text messages. Observations of the frequency of handlings per shift, use of assistive devices, assistance from others, and barriers (interruptions and impediments) were estimated for each worker, aggregated at ward level, and entered into a latent profile analysis, identifying ward phenotypes. We then used generalized estimating equations to determine associations between ward phenotypes, musculoskeletal pain and sickness absence.

**Results:**

We identified four ward phenotypes: ‘turbulent’ (many handlings with devices and assistance, many barriers), ‘strained’ (many handlings without devices or assistance, some barriers), ‘unpressured’ (few handlings, yet without devices or assistance, few barriers) and ‘balanced’ (some handlings with devices and assistance, some barriers). Compared to workers in balanced wards, workers in turbulent wards had more days with neck-shoulder and low-back pain (LBP); and those working in strained wards had more days with LBP and higher pain intensities.

**Conclusion:**

We found that ward phenotypes based on resident handling characteristics were predictive of musculoskeletal pain and sickness absence over one year. This shows that organizational factors related to resident handling are important determinants of musculoskeletal health among eldercare workers.

Eldercare workers often report low-back pain (LBP), neck-shoulder pain (NSP) and musculoskeletal sickness absence, likely due to high physical workload and poor psychosocial working conditions (1–3). This is not only an issue for current workers, it also impedes recruitment of a new workforce required to look after the increased number of elderly in need of care in Europe (4, 5).

As proposed in previous exposure–effect models (6, 7), musculoskeletal health at work is determined in a complex interplay between factors pertaining to tasks, jobs, and the work environment (external exposures), and other factors influencing the eventual mechanical load on individual workers (internal exposures such as postures, movement and forces). In eldercare work, resident handlings are a key external exposure in the development of musculoskeletal disorders (8, 9) since they lead to complex and high biomechanical demands, including pronounced arm elevation, trunk flexion and trunk rotation, often while handling large loads (10, 11). This external exposure to handlings is, in turn, influenced by surrounding organizational factors setting conditions for performing the handlings (6). Examples are under-staffed wards, shortage of time to perform tasks as prescribed, and broken assistive devices, all of which can result in compromised resident handling. The resulting internal exposure on the individual worker gives rise to acute responses (eg, increases in oxygen consumption, heart rate, and perceived exertion), which may, in the long term, have effects on musculoskeletal health (6). Several aspects of handlings – such as their frequency, possible interruptions, and use (or not) of assistive devices – may influence the eventual internal exposure and thus musculoskeletal health (11). For instance, failure to follow handling guidelines can negatively influence musculoskeletal health (12–14). Since handling aspects occur in parallel, they should be addressed together, instead of one at a time, as in previous eldercare studies (15, 16).

To a major extent, previous studies in eldercare have also been based on self-reported data on physical workload, which are prone to bias (17). As an alternative, observational exposure assessment tools have been developed to measure physical workloads during caretaking (18–21). However, to our knowledge, onsite observations have not been applied in any study addressing effects on health of combined handlings characteristics in eldercare.

Eldercare homes are often organized in separate wards, differing in organizational, managerial and structural characteristics that likely influence resident handlings and consequently the musculoskeletal health of workers at the wards. Therefore, examining handlings at a ward level would allow a better understanding of the organizational determinants of exposures, which, in turn, could lead to evidence-based interventions at that level, eg, addressing staff ratio or the availability of assistive devices. Such interventions would likely be more effective and sustainable than initiatives focusing solely on the behaviors of the individual worker (eg, personal training programs), which are known to have limited effectiveness (22). In the present study, we combine the need to address several handling aspects in parallel with the idea of acting at the ward level by determining latent sub-populations of wards sharing similar resident handling characteristics, using latent profile analysis. We label these sub-populations as ward ‘phenotypes’, referring to the term used in genetics for *a set of observable characteristics of an organism* (23).

This study aims to identify phenotypes of wards sharing similar physical and psychosocial conditions for resident handlings and then determine the extent to which they are associated with musculoskeletal pain and sickness absence among workers at the wards over the course of one year.

## Methods

### Design and study population

This prospective study uses data from the Danish Observational Study of Eldercare work and musculoskeletal disorders (DOSES), collected between September 2013 and January 2016 and previously described (24). Briefly, DOSES is a prospective study, in which data at multiple organizational levels were considered. In total 553 eldercare workers, employed in 126 wards nested in 20 eldercare homes, were included with the following inclusion criteria: aged 18–65 years; employed >15 hours/week; working on day, evening or rotating shifts (ie, not exclusively at night); and spending ≥25% of their working time in tasks associated with actual resident care. The Danish Data Protection Agency and the regional Ethics Committee in Copenhagen, Denmark, (H-4-2013-028) approved the study and all participants provided written informed consent to participate. Baseline data collection included questionnaires and onsite observations. During a one-year follow-up, data on self-reported musculoskeletal pain and sickness absence were collected. In our subsequent analyses, we excluded workers who refused to be observed, did not report their work schedules and/or did not answer the questions related to musculoskeletal pain and/or sickness absence at baseline and/or follow-up. To ensure that handlings were representative of wards, we only included wards with ≥2 participating workers. Eventually, the present study included 467 workers distributed across 103 wards in 20 eldercare homes.

### Measurements

#### Descriptive characteristics

At baseline, ward managers were asked to complete a questionnaire addressing conditions at their ward. These ward managers were, in general, responsible for communicating with higher-level management, taking decisions at their own ward, and managing the staff there. The questionnaire asked about type of ward (somatic, dementia, rehabilitation or psychiatric unit), resident information (ie, number of residents in the wards; weight, physical and psychosocial function level of each resident), staff ratio, and how often assistive devices were not in place (daily, 1–3 times/week, 2 times/month or ≤1 time/month). Physical function level was categorized based on the resident’s need for physical assistance (light, moderate, extensive or complete need). Psychosocial function level was categorized based on the resident’s usual behavior (mostly neutral, mostly positive/appreciative, mostly resistant, and mostly aggressive).

The eldercare workers answered a baseline questionnaire asking about age, smoking habits (smoker or non-smoker) and musculoskeletal pain and sickness absence due to NSP and/or LBP, as described in detail below. Body mass index (BMI) was calculated based on measured height and weight (kg/m^2^).

#### Observations – resident handling characteristics

Handling tasks were observed using the DOSES observation instrument, shown previously to have a good reliability (20). At any particular ward, trained research assistants followed workers for 4 hours during day shifts and 4–5 hours during evening shifts, recording, to the extent possible, all handlings of all residents on a tablet, using the Noldus Observer XT 11 software (Noldus, Wageningen, The Netherlands). A previous observational study in Danish eldercare homes (21) showed that more than 70% of all handlings occurred during these periods and thus, to save resources, observations in DOSES were limited to these periods.

The observation instrument was composed of 26 items in four sections: setting and surroundings, manual handling activities, psychosocial interactions with the residents, and barriers (20). Observations of manual handling activities and of barriers were included in the subsequent identification of ward phenotypes. Manual handling activities were classified in terms of whether the worker used an assistive device or not (including the type of device used) and whether the handling was performed by the worker alone, or with the assistance of another person (typically a co-worker or a visitor). Barriers included interruptions and impediments (25) during the care of the resident. An interruption was defined as an event that significantly interrupted the eldercare worker in performing a task (eg, an urgent request for assistance from a co-worker); and an impediment was defined as an obstacle for completing a task requiring some effort (eg, broken or missing assistive device).

Each single observation was registered as an exposure associated with the observed resident. Thus, some residents had more registered observations than others because they were in need of more handlings during a shift. A majority of residents in the 20 eldercare homes were observed (88% in day shifts and 79% in evening shifts). Reasons for not observing a resident could be that (i) the resident was not in need of care or was hospitalized, (ii) the eldercare worker taking care of that resident declined to be followed, and (iii) there was a shortage of research assistants at that point in time. Estimates of handling characteristics for non-observed residents were obtained by imputation. Next, handlings at the level of individual eldercare workers were estimated based on work schedules, which were obtained from the ward managers for all workers, irrespective of whether they participated in observations or not. Schedules were then combined with the data based on handlings at the resident level to give a task-based estimate of the number of handlings per shift for each worker, as well as the associated handling characteristics. In our eventual analysis of phenotypes, we considered (i) the frequency of handlings per shift, and calculated (ii), the proportion of those handlings done without any assistive device and (iii), the proportion of handlings done without any assistance from others. We also considered the number of (iv) interruptions per shift and (v) impediments per shift. We aggregated observations of these five variables at the ward level by averaging the handling exposure estimates of all eldercare workers in each specific ward. In this study, observations during day and evening shifts were summarized in terms of values per shift, disregarding time of day. Then, we used these ward-level scores in a latent profile analysis of handling characteristics.

#### Musculoskeletal pain and sickness absence

The eldercare workers reported musculoskeletal pain and sickness absence at baseline by answering a questionnaire, and during the one-year follow-up by answering, at the most, six questions in text messages (www.sms-track.com) (24, 26). At baseline and every four weeks during follow-up, the workers were asked to report the number of days they experienced NSP and LBP during the preceding four weeks (0–28 days). If participants reported any pain in one or both of these regions, they received a follow-up question about the intensity of their NSP and/or LBP (scale 0–10), and the number of days (0– 28) when pain interfered with work (“Within the past four weeks, how many days did pain in your low-back and/or neck-shoulder made it difficult to perform your normal work, ie, interfered/limited your performance at work?”).

At baseline, and every 12 weeks in follow-up, the workers were also asked to report the number of days on sickness absence due to NSP and/or LBP in the past three months (0–84 days). Answers to pain-related work interference were dichotomized into 0 or ≥1 days, while the other two variables describing number of days were kept continuous.

### Statistical analysis

Based on the handling characteristics of each of the 103 wards (frequency of handlings, proportion of handlings done without assistive devices, proportion of handlings done without assistance from others, number of interruptions, and number of impediments), we performed a latent profile analysis, using the Latent Gold software (version 5.1, Statistical innovations, Belmont, MA, USA). This technique allows multiple variables to be combined in an analysis identifying latent profiles, ie, subpopulations within a population sharing similar properties with respect to the set of observed variables (27–29). In our case, the latent profile analysis was used to identify wards sharing handling characteristics, as a basis for identifying ‘phenotypes’ of wards (see below), which could then be examined for their ability to predict musculoskeletal pain and sickness absence.

The latent profile analysis requires the user to decide the number of profiles that suits data the best. In this process, we considered model fit indices as well as the interpretability of identified profiles. Model fit statistics included the Akaike information criterion (AIC) and the Bayesian information criterion (BIC), showing the trade-off between the simplicity of the model and the goodness of fit, and entropy, describing the degree of certainty of the classification. We also required each profile to contain at least five wards so that even the smallest profile would be generalizable. To better understand the distinct handling characteristics of the eventually identified profiles, we compared the characteristics of wards, workers and residents in each profile, using analysis of variance (ANOVA) for descriptive continuous variables and Chi-squared tests for descriptive categorical variables. At this point, and after further examining the specific ward characteristics expressed in each profile, we labeled the ward profiles as phenotypes, in the sense that they reflect a set of observable and interpretable characteristics pertaining to the ward.

To estimate the effects of working at a certain ward phenotype (ward-level predictor) on reported musculoskeletal pain and sickness absence during the one-year follow-up (individual-level outcome) we used generalized estimating equations (GEE) (30). In these analyses, each worker was assigned the ward phenotype in which they worked as a predictor, and their self-reported musculoskeletal pain and sickness absence at each time point during follow-up as the outcome (14 time points for pain and 5 time points for sickness absence). We included time as a within-subject covariate, and workers and wards as random effects in an unstructured correlation matrix. For the continuous outcome variables (days with NSP, intensity NSP, days with LBP, intensity LBP, and sickness absence due to NSP/LBP), we used a Poisson distribution model, with a log link function. For the dichotomous variable (pain-related work interference), we used a binary logistic model.

We adjusted each of the six models for their corresponding outcome values obtained at baseline. We further adjusted the models for age, BMI and smoking habits. We also did a sensitivity analysis by re-running each of the six primary models solely with participants reporting ‘little’ pain at baseline, defined as pain ≤5 days/month, pain intensity ≤2 (0–10 scale), 0 days with pain-related work interference, and 0 days with sickness absence. We performed all tests in SPSS (Statistical Package for Social Science, v. 20, IBM Corp, Armonk, NY, USA) and set the significance level at 0.05.

## Results

### Identification and characterization of ward phenotypes

According to the model fit statistics (supplementary material, https://www.sjweh.fi/article/3979, table S1), models with four and five profiles were the more certain (higher entropy values), while still being sufficiently parsimonious according to BIC and AIC values. These two models also fulfilled the criterion of the smallest profile containing at least five wards. Taking into account that the model with four profiles showed better parsimony and had more workers in the smallest profile than the model with five profiles, and that the differences in the handling characteristics between the four ward profiles were more intuitive than those in the five-profiles model, we settled on the four-profile solution.

**Figure 1:**
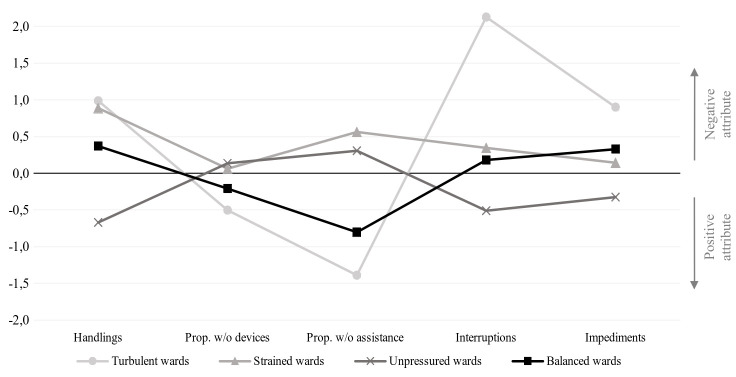
Standardized values (Z-scores) of number of handlings per shift, proportion of handlings without assistive devices (proportion w/o devices), proportion of handlings without assistance from co-workers or others (prop. w/o assistance), number of interruptions per shift and number of impediments per shift, in each ward phenotype. Each phenotype was identified using a latent profile analysis based on observed handling characteristics at the ward level. Positive values in the y-axis represent negative attributes, ie, higher number of handlings, higher proportion of handlings done without devices, higher proportion of handlings done without help and higher rate of interruptions and impediments per shift.

We then examined the four ward profiles (see figure 1) and described their properties in terms of four phenotypes with the following labels:

***‘Turbulent’ wards***: many handling operations (mean 11.8, standard deviation (SD) 7.1 per shift], some of which are done without assistive devices [mean 41 (SD 18) %] or assistance from others [mean 20 (SD 9) %], and with many barriers [mean 6.2 (SD 2.5) interruptions per shift; mean 2.7 (SD 1.8) impediments per shift].

***‘Strained’ wards:*** many handling operations [mean 11.4 (SD 4.3) per shift], many of which are done without assistive devices [mean 52 (SD 18) %] or assistance from others [mean 67 (SD 10) %], with some barriers [mean 3.1 (SD 1.8) interruptions per shift; mean 1.9 (SD 1.3) impediments per shift].

***‘Unpressured’ wards***: few handling operations [mean 4.9 (SD 1.8) per shift], even if many of which are done without assistive devices [mean 53 (SD 21) %] or assistance from others [mean 61 (SD 24) %], and few barriers [mean 1.7 (SD 0.8) interruptions per shift; mean 1.5 (SD 0.7) impediments per shift].

***‘Balanced’ wards***: some handling operations [mean 9.2 (SD 1.6) per shift], some of which without assistive devices [mean 47 (SD 12) %] or assistance from others [mean 34 (SD 11) %], and with some barriers [mean 2.9 (SD 1.0) interruption per shift; mean 2.1 (SD 0.7) impediments per shift].

Based on the latent profile analysis, mean 7 (6.8%), 21 (20.4%), 52 (50.5%) and 23 (22.3%) of the 103 wards were categorized as turbulent, strained, unpressured, and balanced, respectively. Table 1 presents descriptive statistics on wards, workers and residents, stratified by ward phenotype.

**Table 1:** Descriptive characteristics at ward, worker and resident level for each ward phenotype (N=103), as identified using latent profile analysis of observed handling characteristics, and P-values for differences between ward phenotypes. Data based on the baseline questionnaire. [SD=standard deviation.]

	Total		Turbulent wards		Strained wards		Unpressured wards		Balanced wards		P-value
				
	Mean (SD)	N (%)	Mean (SD)	N (%)	Mean (SD)	N (%)	Mean (SD)	N (%)	Mean (SD)	N (%)	
Ward characteristics											
Staff ratio	0.46 (0.10)		0.42 (0.16)		0.44 (0.08)		0.47 (0.12)		0.47 (0.07)		**0.01**
Type of ward											**<0.01**
Somatic		77 (76.2)		6 (85.7)		21 (100.0)		36 (70.6)		14 (63.6)	
Dementia		20 (19.6)		1 (14.3)		0 (0.0)		13 (25.5)		6 (27.3)	
Rehabilitation		2 (1.9)		0 (0.0)		0 (0.0)		2 (3.9)		0 (0.0)	
Psychiatric		2 (1.9)		0 (0.0)		0 (0.0)		0 (0.0)		2 (9.1)	
Devices not in place											**<0.01**
Daily		9 (11.8)		0 (0.0)		6 (31.6)		1 (2.7)		2 (11.8)	
1–3 times/week		2 (2.6)		0 (0.0)		0 (0.0)		2 (5.4)		0 (0.0)	
2 times/month		13 (17.1)		1 (33.3)		3 (15.8)		8 (21.6)		1 (5.9)	
≤1 time/month		51 (68.0)		2 (66.7)		10 (52.6)		26 (70.3)		14 (82.4)	
Worker characteristics											
Number of workers		467 (100.0)		23 (4.9)		74 (15.8)		251 (53.7)		119 (25.5)	
Age (years)	45.5 (10.7)		45.0 (10.6)		44.5 (11.5)		45.1 (10.3)		46.8 (11.3)		0.38
Body mass index (kg/m^2^)	26.4 (5.3)		25.0 (5.1)		27.8 (5.7)		26.5 (5.3)		25.5 (4.7)		**0.01**
Smoking habits											0.46
Smokers		135 (28.9)		8 (34.8)		22 (29.7)		71 (28.3)		34 (28.6)	
Non-smokers		332 (71.1)		15 (65.2)		52 (70.3)		180 (71.7)		85 (71.4)	
Resident characteristics											
Number of residents		1112 (100.0)		76 (6.8)		212 (19.1)		585 (52.6)		239 (21.5)	
Weight (kg) ^a^	65.6 (5.2)		64.1 (2.3)		65.2 (5.0)		65.5 (4.1)		66.7 (7.9)		0.87
Physical function level ^a^											**<0.01**
Light		243 (21.9)		18 (23.7)		36 (17.0)		140 (23.9)		49 (20.5)	
Moderate		379 (34.1)		21 (27.6)		55 (25.9)		218 (37.3)		85 (35.6)	
Extensive		222 (20.0)		19 (25.0)		53 (25.0)		106 (18.1)		44 (18.4)	
Completely		268 (24.1)		18 (23.7)		68 (32.1)		121 (20.7)		61 (25.5)	
Psychosocial function level ^a^											0.30
Mostly neutral		313 (28.3)		22 (28.9)		73 (35.1)		162 (27.7)		56 (23.4)	
Mostly appreciative		429 (38.8)		26 (34.2)		78 (37.5)		224 (38.4)		101 (42.3)	
Mostly resistant		275 (24.8)		22 (28.9)		42 (20.2)		146 (25.0)		65 (27.2)	
Mostly aggressive		90 (8.1)		6 (7.9)		15 (7.2)		52 (8.9)		17 (7.1)	

a Information provided by team managers. Bold values show significant differences between phenotypes.

At turbulent wards, the staff ratio was smaller than at the other ward phenotypes and a higher proportion of residents were classified as needing complete physical assistance. At strained wards, devices were reported to be misplaced daily more often than in the other ward phenotypes, and the workers had higher BMI. Unpressured and balanced wards were not extreme in any respect. Descriptive characteristics of the eldercare workers’ musculoskeletal pain and sickness absence at baseline and during the one-year follow-up, stratified by ward phenotype, are shown in table 2.

**Table 2:** Musculoskeletal pain and sickness absence at baseline and over the one-year follow-up for the four ward phenotypes. Each phenotype was identified using a latent profile analysis based on observed handling characteristics at ward level. Values are presented as mean (standard deviation) between workers within a specific phenotype. [SD=standard deviation; LBP=low-back pain; NSP=neck-shoulder pain].

	Turbulent wards	Strained wards	Unpressured wards	Balanced wards
			
	Mean (SD)	Mean (SD)	Mean (SD)	Mean (SD)
Days w/ NSP/month (0–28 days)				
Baseline	4.9 (8.3)	9.1 (9.8)	8.4 (10.1)	7.8 (9.8)
During follow-up	8.2 (8.0)	8.9 (7.8)	7.3 (7.6)	6.7 (6.6)
Intensity NSP/month (0–10 scale)				
Baseline values	3.4 (3.4)	4.4 (4.4)	4.1 (3.2)	3.8 (3.1)
During follow-up	3.5 (2.1)	4.2 (2.4)	3.5 (2.5)	3.2 (2.5)
Days w/ LBP/month (0–28 days)				
Baseline values	5.3 (9.2)	8.0 (8.5)	7.9 (9.5)	7.8 (9.7)
During follow-up	8.6 (8.5)	7.9 (7.0)	7.0 (7.3)	5.9 (6.5)
Intensity LBP/month (0–10 scale)				
Baseline values	3.5 (3.4)	4.3 (3.2)	4.0 (3.2)	3.8 (3.2)
During follow-up	3.8 (2.4)	4.1 (2.5)	3.4 (2.4)	3.1 (2.5)
Days w/ pain-related work interference (0–28 days)				
Baseline values	2.9 (6.3)	3.5 (5.8)	4.1 (7.8)	3.8 (7.4)
During follow-up	6.7 (6.3)	5.7 (6.3)	4.8 (6.3)	3.9 (4.9)
Days w/ musculoskeletal sickness absence (0–84 days)				
Baseline values	0.2 (0.7)	0.6 (2.1)	1.1 (6.4)	1.1 (5.5)
During follow-up	3.4 (9.3)	2.1 (5.7)	1.8 (5.8)	0.9 (3.0)

### Associations between ward phenotypes, musculoskeletal pain and sickness absence

During the one-year follow-up, turbulent wards were significantly associated with more days per month with NSP (β=0.54; P<0.01) and LBP (β=0.37; P<0.01) among the workers than balanced wards (table 3). Similar associations were found after adjustments for age, BMI and smoking habits, although only statistically significant for LBP (β=0.35; P=0.01). Thus, the adjusted model predicted that the number of days with LBP per month at follow-up would be 0.35 days larger at turbulent versus balanced wards.

**Table 3:** Associations between ward phenotypes, musculoskeletal pain and sickness absence over the one-year follow-up (14 time points for pain and 5 for sickness absence). Each phenotype (turbulent, strained, unpressured, and balanced) was identified using a latent profile analysis based on observed handling characteristics at ward level. Balanced wards was used as reference in all analyses. **Bold indicates a statistically significant difference.** [CI=confidence interval].

Ward phenotypes	Model 1^a^			Model 2 ^b^		
	
	Coefficient	95% CI	P-value	Coefficient	95% CI	P-value
Days with NSP per month (0–28 days) ^c^						
Turbulent	0.54	0.26–0.82	**<0.01**	0.46	-0.21–1.13	0.18
Strained	0.17	-0.05–0.38	0.12	0.17	-0.30–0.64	0.48
Unpressured	0.04	-0.13–0.22	0.65	0.14	-0.24–0.52	0.47
Balanced	0.00			0.00		
Intensity NSP per month (0–10 scale) ^c^						
Turbulent	0.12	-0.07–0.30	0.21	0.15	-0.04–0.34	0.13
Strained	0.14	0.01–0.28	**0.03**	0.18	0.04–0.31	**0.01**
Unpressured	0.04	-0.07–0.16	0.46	0.06	-0.06–0.19	0.32
Balanced	0.00			0.00		
Days with LBP per month (0–28 days) ^c^						
Turbulent	0.37	0.16–0.58	**<0.01**	0.35	0.08–0.63	**0.01**
Strained	0.21	0.04–0.39	**0.02**	0.22	0.03–0.42	**0.03**
Unpressured	0.08	-0.07–0.22	0.29	0.11	-0.05–0.26	0.17
Balanced	0.00			0.00		
Intensity LBP per month (0–10 scale) ^c^						
Turbulent	0.17	-0.05–0.40	0.13	0.11	-0.13–0.35	0.35
Strained	0.15	0.01–0.29	**0.04**	0.17	0.02–0.31	**0.03**
Unpressured	0.03	-0.08–0.13	0.62	0.05	-0.06–0.16	0.41
Balanced	0.00			0.00		
Days musculoskeletal sickness absence (0–84 days) ^c^						
Turbulent	0.86	-0.28–2.01	0.14	0.90	-0.21–2.02	0.11
Strained	0.83	-0.05–1.71	0.07	0.67	-0.25–1.60	0.15
Unpressured	0.40	-0.30–1.11	0.26	0.34	-0.38–1.06	0.35
Balanced	0.00			0.00		
Days with pain-related work interference – categorical (% with ≥1 days/month) ^d^						
Turbulent	1.53	0.83–2.83	0.17	1.33	0.67–2.63	0.40
Strained	1.67	1.14–2.44	**0.01**	1.79	1.20–2.66	**<0.01**
Unpressured	1.16	0.88–1.54	0.29	1.20	0.89–1.61	0.22
Balanced	1.00			1.00		

a Adjusted for the baseline values of the outcomes.

b Further adjusted by age, body mass index, smoking habits.

c Model coefficient value expressed as ß.

d Model coefficient value expressed as odds ratio.

Strained wards were associated with higher intensity of NSP pain (β=0.14; P=0.03), more days with LBP (β=0.21; P=0.02), and higher intensity of LBP (β=0.15; P=0.04) and were more likely to report pain-related work interference [odds ratio (OR) 1.67; P=0.01] compared with balanced wards. Strained wards were also associated with more days with musculoskeletal sickness absence (β=0.83; P=0.07) than balanced wards. After adjustments these results remained significant and with slightly higher effect estimates (table 3). Unpressured wards did not differ significantly from balanced wards in pain and sickness absence.

The sensitivity analysis of workers with low pain and no sickness absence at baseline showed, in general, stronger and more significant associations at follow-up in this sub-population than in the total population (supplementary table S2).

## Discussion

### Summary of findings

To our knowledge, our study is the first to collect onsite observations of resident handlings in eldercare, interpret those in terms of ward phenotypes, and address the prospective association of these phenotypes with musculoskeletal pain and sickness absence among eldercare workers. We identified four distinct ward phenotypes, labeled as turbulent, strained, unpressured, and balanced, representing distinctly different combinations of various handling characteristics. After adjusting for age, BMI and smoking habits, we found that workers at turbulent and strained wards reported a larger increase in pain during the one-year follow up than workers at balanced wards.

### Identified ward phenotypes

Among the four ward phenotypes, two (turbulent and strained) appeared to have a less favorable combination of handling characteristics, while the two others (unpressured and balanced) had more beneficial profiles. To our knowledge, this is the first study to evaluate different aspects of resident handlings concomitantly.

At turbulent wards, workers had high physical demands reflected by a high frequency of handlings, but they also had resources to perform these handlings, as shown by the high proportion of handlings done with assistive devices and assistance from others. Handling assistance has been shown to reduce physical load during handling tasks (15, 31). On the other hand, handlings at turbulent wards also suffered many barriers, which has been associated with poor health (21, 25). Turbulent wards had a low staff ratio compared to other ward phenotypes (table 1), and the high number of interruptions may be explained by workers being disturbed more often because their colleagues needed assistance.

The strained wards also appear to have less favorable handlings characteristics; handlings were both frequent and, to a considerable extent, performed without assistive devices or assistance from others, likely have increasing the physical load on workers (15, 16, 31). Unpressured and balanced wards showed more favorable handling characteristics, even though they were not ideal. Based on results from previous studies, ‘ideal’ wards would have <10 handlings per shift (11), most of these performed with assistive devices or assistance from others (15, 16, 31), and only few barriers (21, 25). Our current sample of wards did not include any such ideal ward phenotype. While interesting, a further examination of the determinants of ward-level handling characteristics, and hence of the handling phenotypes, was beyond the aims of the present study.

### Association of ward phenotypes with musculoskeletal pain and sickness absence

Compared with balanced wards, working in a turbulent ward was associated with more frequent NSP and LBP, while working in a strained ward was associated with more days of LBP, higher intensity of NSP and LBP and a higher likelihood of pain interfering with work. This suggests that the risk of musculoskeletal pain increases with the frequency of handlings, particularly when handlings are more often performed without the use of assistive devices or assistance from others. This is consistent with previous studies reporting that a higher frequency of handlings is associated with persistent LBP in eldercare workers (11) and a higher risk of back injuries among hospital healthcare workers (32). Notably, these previous studies, like several others (12–14, 31, 33), considered just one aspect of handlings at the time and were focused on individual-based exposure assessment. Previous studies suggest that pain intensity and pain-related work interference have clear associations with sickness absence among healthcare workers (34). But we did not find a statistically significant association between sickness absence and the ward phenotypes, even though we observed a tendency for more absence days due to musculoskeletal pain among workers at strained wards.

Performing resident handlings without assistive devices, as observed to a larger extent in strained wards, have been associated with LBP among nurses (35), but evidence supporting the efficacy of using assistive devices in reducing musculoskeletal pain among healthcare workers is still limited (36). A possible explanation is that studies often consider the use of assistive devices as an isolated factor and ignore other important organizational factors that may act in parallel and correlate with the use of devices. By including job barriers (interruptions and impediments), defined as poor organizational, psychosocial and/or physical work conditions impeding the performance of work tasks (20, 37, 38), we addressed factors beyond the mere physical aspects of handlings. Thus, our ward phenotypes reflect a set of important external exposures associated with resident handlings, and our study considers the eventual results of these exposures in terms of individual outcomes, ie, pain and sickness absence, as illustrated in the exposure-effect model (6). A previous study showed that interruptions and impediments assessed using onsite observations were associated with musculoskeletal disorders in urban transit operators (25), but to our knowledge no previous study has examined this association among eldercare workers.

We found a large variability in reported pain and sickness absence at baseline, and so we also performed a sensitivity analysis including only those workers with little pain and no sickness absence. Ward phenotype predicted musculoskeletal pain and sickness absence even stronger in this sub-population, suggesting that the handling characteristics at turbulent and strained wards could, indeed, increase the risk of workers developing musculoskeletal disorders.

### Methodological considerations, implications and future research

The use of a systematic and reliable onsite observation tool for assessing resident handlings characteristics (20, 24) is a strength of this study as it provided information that will likely be more accurate than that obtained, eg, by self-reports from the workers. Also, this approach eliminates the risk of common method bias, which is a likely fallacy in studies where both exposures and outcomes are based on self-report (17). In aggregating observations at the ward level, thus obtaining ‘non-individualized’ information about general handling characteristics at a specific ward, we further reduced the risk of common method bias. Another strength of the study is the novel use of latent profile analysis, which allowed us to identify clusters of wards sharing similar combinations of handling characteristics, which we could then interpret and label in terms of ward phenotypes. The use of latent profile analysis also allowed us to integrate physical and psychosocial aspects of handlings, and thus to examine their combined impact on musculoskeletal health. We believe that ‘clustered’ exposure assessment approaches like this offer an appealing alternative to standard multivariate regression, where exposure variables are entered in parallel, and correlations may severely challenge the interpretation of results. The extensive assessment of pain and sickness absence, using 14 and 5 repeated measurements, respectively, may also be considered a strength of the study.

Still, the study also suffers some limitations. Even though the use of an observational tool can be considered an advantage compared to self-reports, it should ideally be combined with direct measurements of exposures (39), which we did not. Observations at a particular ward were performed during a short period of time, and the estimated handling variables at the ward level are subject to some inaccuracy due to variability between days, both on the short term, for instance because residents have different needs on different days, and on the longer term, because residents may be replaced by other residents with different needs, or staffing may change. Also, handlings for, on average, about 15% of the residents at a ward were obtained by imputation, which may have introduced additional variance. However, based on our experiences in eldercare, we believe these sources of exposure variability to be moderate, and thus having only a minor impact on the accuracy of the average ward-level exposure. Notably, our exposure assessment strategy was not sensitive to the proportion of participating workers, since work schedules were obtained for everybody, directly from the ward manager (24). Ward phenotypes were determined in a data-driven process, and even though our phenotypes appear representative of eldercare conditions, it is possible that the ward classification could be different in other populations. Future studies are needed to verify the stability of the phenotyping across wards in general.

The assessment of pain and sickness absence during the one-year follow-up relied solely on self-reports, which could introduce bias. However, we trust this risk to be reduced by the frequent assessment of outcomes, reducing recall bias, and we emphasize that common method variance of exposures and outcomes is likely small, due to the use of observational-based, aggregated exposures. Finally, while we believe each of the six selected outcomes to be important in reflecting different aspects of pain (severity, duration) and its consequences (interference with work, sickness absence due to musculoskeletal pain), we acknowledge the risk of obtaining significant results by chance in multiple tests.

Our results, in illustrating the importance of ward-level characteristics to health can be used in future studies as a basis for designing ward-level tools for preventing musculoskeletal symptoms in eldercare workers, as an alternative to, or complementing, individualized interventions, such as having workers attend courses in lifting techniques. While we believe that intervening on organizational and structural determinants of handlings would lead to more effective and sustainable results than interventions directed towards the behaviors of individual workers, future studies should verify the effectiveness of ward-level organizational tools, for example in randomized control trials, before they are generally recommended for practice (40).

### Concluding remarks

Based on onsite observations of resident handlings in eldercare, we were able to identify four handling phenotypes at the ward level. Our results show that turbulent and strained ward phenotypes were associated with some indicators of musculoskeletal pain during the one-year follow-up. Thus, our study suggests that ward phenotypes with frequent handlings, either combined with limited use of assistive devices or with many barriers, may impose an increased risk of musculoskeletal pain among the workers. Our findings encourage future studies devoted to the development of ward-level, organizational interventions in eldercare homes.

## Supplementary material

Supplementary material
