# Supplementary material for: Are resident handlings in eldercare wards associated with musculoskeletal pain and sickness absence among the workers? A prospective study based on onsite observations
Source: Scand J Work Environ Health. 2021 Oct 31;47(8):609–18. doi: 10.5271/sjweh.3979 (PMC9058617; doi:10.5271/sjweh.3979)
Supplement: Supplementary material [file SJWEH-47-609-S001.pdf]

Are resident handlings in eldercare wards associated with musculoskeletal pain and sickness absence among the workers? A prospective study based on onsite observations[1]

by Leticia Bergamin Januario, PhD,[2] Svend Erik Mathiassen PhD, Matthew L Stevens, PhD, Andreas Holtermann, PhD, Gunnar Bergström, PhD, Reiner Rugulies, PhD, Kristina Karstad, PhD, David M Hallman, PhD

1. *Supplementary material*
2. *Correspondence to: Leticia Bergamin Januario, Department of Occupational Health Sciences and Psychology, Centre for Musculoskeletal Research, University of Gävle Kungsbäcksvägen 47, 801 76, Gävle, Sweden. [E-mail: leticia.januario@hig.se]*

Table S1. Model fit statistics in analyses identifying from 1 to 5 latent profiles, based on workplace observations regarding resident handling characteristics in 103 wards and 467 eldercare workers.

	BIC	AIC	Entropy	N_{wards}	$N_{workers}$
One profile	1282.3	1255.8	-	-	-
Two profiles	1243.4	1187.8	0.68	40	173
Three profiles	1267.5	1182.9	0.70	4	14
Four profiles	1300.1	1186.4	0.77	7	23
Five profiles	1331.3	1188.5	0.81	6	16

AIC: Akaike Information Criterion (lower value, better fit); BIC: Bayesian Information Criterion (lower value, better fit); Entropy (higher value, better fit), N: number of wards and workers in the smallest profile.

Table S2. Sensitivity analysis, considering workers with low pain and no sickness absence at baseline (for frequency of pain the threshold was considered ≤5 days/month and for intensity ≤2 in 0-10 scale). Adjusted associations between ward phenotypes, musculoskeletal pain and sickness absence over the one-year follow-up (14 time points for pain and 5 for sickness absence). Each phenotype (‘Turbulent’, ‘Strained’, ‘Unpressured’ and ‘Balanced’) was identified using a latent profile analysis based on observed handling characteristics at ward level.

		Model 1[a]			Model 2 [b]		
	N wards (N workers)	Coefficient	95% CI	P value	Coefficient	95% CI	P value
Days with NSP per month (0-28 days) [c]							
‘Turbulent’ wards	7 (19)	1.96	1.48 – 2.43	**<0.01**	1.03	-0.17 – 2.23	0.09
‘Strained’ wards	10 (39)	0.08	-0.45 – 0.30	0.69	0.43	-0.76 – 1.62	0.48
‘Unpressured’ wards	31 (153)	-0.67	-1.03 – -0.31	**<0.01**	-0.25	-1.38 – 0.88	0.66
‘Balanced’ wards	15 (72)	0.00	–	–	0.00	–	–
Intensity NSP per month (0-10 scale) [c]							
‘Turbulent’ wards	6 (12)	0.54	-0.05 – 1.14	0.07	0.40	-0.44 – 0.63	0.44
‘Strained’ wards	6 (25)	0.35	-0.64 – 0.71	0.92	-0.08	-1.02 – 0.87	0.87
‘Unpressured’ wards	18 (89)	-1.34	-2.50 – -0.18	**0.02**	-1.58	-2.60 – -0.55	**<0.01**
‘Balanced’ wards	10 (44)	0.00	–	–	0.00	–	–
Days with LBP per month (0-28 days) [c]							
‘Turbulent’ wards	5 (18)	1.09	0.09 – 2.09	**0.03**	1.81	0.04 – 3.59	**0.05**
‘Strained’ wards	11(39)	0.11	-1.19 – 1.40	0.87	1.01	-0.75 – 2.78	0.26
‘Unpressured’ wards	30 (144)	-0.68	-1.93 – 0.57	0.29	0.41	-1.37 – 2.18	0.65
‘Balanced’ wards	13 (71)	0.00	–	–	0.00	–	–

Intensity LBP per month (0-10 scale) [c]							
‘Turbulent’ wards	2 (11)	0.94	0.29 – 1.58	**<0.01**	0.87	0.22 – 1.52	**0.01**
‘Strained’ wards	7 (23)	0.71	0.12 – 1.30	**0.02**	0.85	0.26 – 1.44	**<0.01**
‘Unpressured’ wards	18 (99)	0.58	0.11 – 1.05	**0.02**	0.60	0.18 – 1.02	**0.01**
‘Balanced’ wards	11 (45)	0.00	–	–	0.00	–	–
Days musculoskeletal sickness absence (0-84 days) [c]							
‘Turbulent’ wards	7 (21)	1.59	0.24 – 2.95	**0.02**	1.65	0.28 – 3.03	**0.02**
‘Strained’ wards	16 (61)	1.34	0.26 – 2.43	**0.01**	1.20	0.09 – 2.31	**0.03**
‘Unpressured’ wards	43 (208)	0.86	-0.12 – 1.84	0.09	0.79	-0.25 – 1.84	0.14
‘Balanced’ wards	21 (104)	0.00	–	–	0.00	–	–
Days with pain-related work interference, categorical (% with ≥1 days over a month) [d]							
‘Turbulent’ wards	3 (11)	1.70	0.79 – 3.36	0.18	1.60	0.64 – 4.04	0.32
‘Strained’ wards	11 (40)	1.60	0.99 – 2.58	**0.05**	1.84	1.11 – 3.05	**0.02**
‘Unpressured’ wards	36 (152)	1.09	0.77 – 1.55	0.62	1.15	0.79 – 1.67	0.46
‘Balanced’ wards	16 (72)	1.00	–	–	1.00	–	–

a: adjusted for the baseline values of the outcomes; b: further adjusted by age, BMI, smoking habits; c: model coefficient value expressed as β; d: model coefficient value expressed as odds ratio. CI: confidence interval (lower bound - upper bound). ‘Balanced’ wards was used as reference in all analyses. Bold values mark a statistically significant difference.
